# Roles of the Neuron-Restrictive Silencer Factor in the Pathophysiological Process of the Central Nervous System

**DOI:** 10.3389/fcell.2022.834620

**Published:** 2022-03-01

**Authors:** Xin-Jin Su, Bei-Duo Shen, Kun Wang, Qing-Xin Song, Xue Yang, De-Sheng Wu, Hong-Xing Shen, Chao Zhu

**Affiliations:** ^1^ Department of Spine Surgery, School of Medicine, Renji Hospital, Shanghai Jiao Tong University, Shanghai, China; ^2^ Department of Spine Surgery, School of Medicine, Shanghai East Hospital, Tongji University, Shanghai, China

**Keywords:** neuron-restrictive silencer factor (NRSF), neuron-restrictive silencer element, brain disorders, neuropathic pain, neurogenesis, tumorigenesis

## Abstract

The neuron-restrictive silencer factor (NRSF), also known as repressor element 1 (RE-1) silencing transcription factor (REST) or X2 box repressor (XBR), is a zinc finger transcription factor that is widely expressed in neuronal and non-neuronal cells. It is a master regulator of the nervous system, and the function of NRSF is the basis of neuronal differentiation, diversity, plasticity, and survival. NRSF can bind to the neuron-restrictive silencer element (NRSE), recruit some co-repressors, and then inhibit transcription of NRSE downstream genes through epigenetic mechanisms. In neurogenesis, NRSF functions not only as a transcriptional silencer that can mediate the transcriptional inhibition of neuron-specific genes in non-neuronal cells and thus give neuron cells specificity, but also as a transcriptional activator to induce neuronal differentiation. Many studies have confirmed the association between NRSF and brain disorders, such as brain injury and neurodegenerative diseases. Overexpression, underexpression, or mutation may lead to neurological disorders. In tumorigenesis, NRSF functions as an oncogene in neuronal tumors, such as neuroblastomas, medulloblastomas, and pheochromocytomas, stimulating their proliferation, which results in poor prognosis. Additionally, NRSF-mediated selective targets gene repression plays an important role in the development and maintenance of neuropathic pain caused by nerve injury, cancer, and diabetes. At present, several compounds that target NRSF or its co-repressors, such as REST-VP16 and X5050, have been shown to be clinically effective against many brain diseases, such as seizures, implying that NRSF and its co-repressors may be potential and promising therapeutic targets for neural disorders. In the present review, we introduced the biological characteristics of NRSF; reviewed the progress to date in understanding the roles of NRSF in the pathophysiological processes of the nervous system, such as neurogenesis, brain disorders, neural tumorigenesis, and neuropathic pain; and suggested new therapeutic approaches to such brain diseases.

## Introduction

The neuron-restrictive silencer factor (NRSF), also known as repressor element 1 (RE-1) silencing transcription factor (REST) or X2 box repressor (XBR), is a zinc finger transcription factor that is widely expressed in both neuronal and non-neuronal cells in different species as well as in normal and abnormal brain tissues (Zhao et al., 2017). It was initially reported independently by two study groups in 1995 to be a master repressor in neurogenesis (Schoenherr and Anderson, 1995a; Chong et al., 1995). NRSF inhibits the expression of target genes by binding to the neuron-restrictive silencer element (NRSE/RE-1) that is present in the regulatory region of the neuron-specific genes (Roopra et al., 2004; Valouev et al., 2008; Song et al., 2015). NRSF can also specifically activate neuron-related genes, mainly small non-coding region genes, such as the dynamin I gene (Yoo et al., 2001). The dynamic expression and variable levels of NRSF in different cells and tissues and at different stages throughout the development of the nervous system are vital to blocking the expression of neuron-specific genes in non-neuron cells and ensuring the establishment of neuronal specificity (Palm et al., 1998).

In recent decades, accumulating evidence has also shown the high involvement of NRSF in neurogenesis, brain disorders, tumorigenesis, as well as NPP. Chen et al. reported that knockdown of NRSF during embryogenesis could lead to brain abnormalities and the premature death of mice (Chen et al., 1998). Additionally, more and more compounds that target NRSF or its co-repressors, such as REST-VP16, X5050, and valproic acid (VPA), seem to be clinically effective against brain diseases, including seizures and NPP (Immaneni et al., 2000; Warburton et al., 2015; Zhao et al., 2017). These findings suggest that NRSF plays multiple roles in the pathophysiological process of the nervous system, and it may be a promising potential therapeutic target for certain brain disorders. Therefore, in the current review, we aimed to summarize the recent studies about NRSF in the nervous system, which will facilitate a better understanding of the pathophysiology of NRSF in the nervous system and promote NRSF-targeted clinical applications.

## Biological Characteristics of NRSF

NRSE is a 21- to 23-bp DNA sequence that is highly conserved among species and is the target sequence for NRSF protein binding (Thiel et al., 1998). It was first identified in the voltage-gated type II sodium channel and the promoter of the superior cervical ganglion gene 10 (SCG10) (Kraner et al., 1992). NRSE may not be the only target sequence for NRSF. Otto et al. (2007) and (Johnson et al., 2010) found a bisect sequence that was different from the recognized common sequence of NRSE, and there were 16–19 bases between the two parts of the sequence, suggesting that there might be more NRSF binding sites.

NRSF is a transcription regulatory protein belonging to the Gli–Kruppel transcription factor family with a molecular weight of 116 ku and a full length of 1,069 amino acids (Schoenherr and Anderson, 1995b; Chong et al., 1995). Previous studies have shown that NRSF amino acid sequences in different species have high homology (Bruce et al., 2004). Generally, NRSF contains nine Cys/His2 zinc finger structures, one DNA-binding domain, one lysine-rich region, one proline-rich region, and two repression domains (N-terminal and C-terminal repressor domains) (Schoenherr and Anderson, 1995a; Chong et al., 1995). Zinc finger structures 2 through 5 mainly play the role of nuclear localization, zinc finger structures 6 to 8 can bind to target sequences, and zinc finger structure 9 has the function of target DNA and RNA recognition (Schoenherr and Anderson, 1995b; Chong et al., 1995). NRSF has different transcripts because of alternative splicing. These transcripts lack the C-terminal repressor structure compared to NRSF. They can antagonize the transcriptional inhibition by NRSF and activate gene transcription (Palm et al., 1998). For example, REST 4, a truncated form of NRSF found in humans or rodents and mainly expressed in neuronal cell or tissues, can act as “anti-silencer” that competitively binds to NRSE, thereby promoting neuron-specific gene expression (Tabuchi et al., 2002). In several pathophysiological processes, such as neurogenesis, neurological disorders, and non-neuronal tumorigenesis, the dynamic balance between REST 4 and NRSF has been reported as playing an important role (Coulson et al., 2000; Yu et al., 2009; Raj et al., 2011).

## Mechanism of Transcriptional Inhibition by NRSF

**FIGURE 1:**
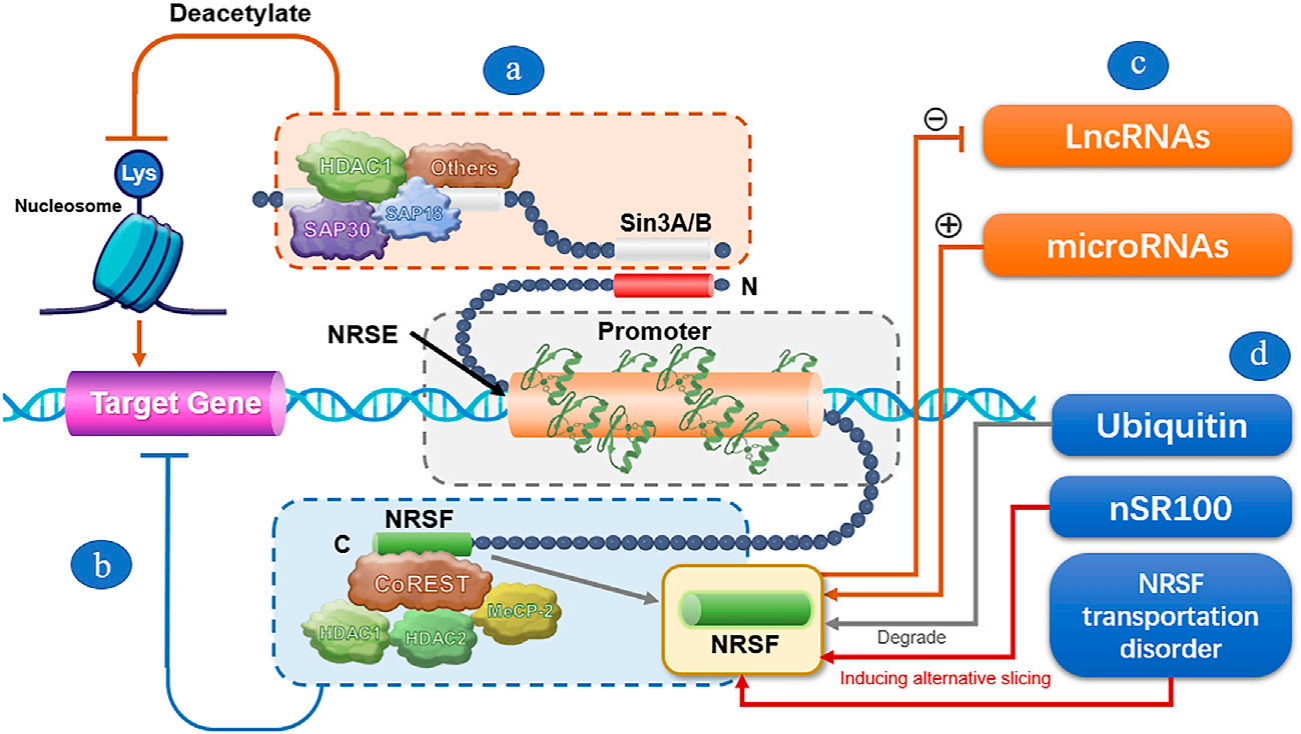
A schematic illustration of the transcriptional repression mechanism by NRSF. **(A)** After specifically binding of NRSF to the NRSE sequence of the target gene, the N-terminal repression domain of NRSF can bind to mSin3A/B, and recruit HDACs and other factors to form transcriptional inhibition complex. The complex deacetylates lysine residues of nucleosome histones, prompting tight nucleosome encapsulation to form heterochromatin that blocks the transcription of target genes and thus maintains gene silencing. **(B)** The C-terminal repression domain can bind to REST co-repressor proteins (CoRESTs), and further attract HDAC1, HDAC2, and MeCP2 to promote and maintain methylated CPG-dependent gene silencing. **(C)** LncRNAs and microRNAs regulate the expression of NRSF through the regulatory feedback mechanism. **(D)** Ubiquitin dynamically modulates NRSF expression by degrading NRSF through ubiquitin-mediated proteolysis. nSR100 negatively regulate NRSF by inducing alternative slicing of NRSF. NRSF transportation disorder reduces the NRSF level in the nucleus, thus alleviating the transcriptional repression by NRSF.

The NRSF-mediated transcriptional inhibition mechanism is very complicated (Figure 1). It is mainly based on repressor domains at both ends of NRSF and relies on the assistance of multiple regulatory factors. The N-terminal and C-terminal repression domains can independently exert transcriptional inhibition of neuron-specific genes by recruiting co-repressors. When NRSF specifically binds to the NRSE sequence of the target gene, the N-terminal repression domain can bind to mSin3A/B and recruit histone deacetylase (HDAC) transcriptional inhibition complexes. The complex deacetylates lysine residues of nucleosome histones, prompting tight nucleosome encapsulation to form heterochromatin that blocks the transcription of target genes and thus maintains gene silencing (Naruse et al., 1999). The C-terminal repression domain can bind to REST co-repressor proteins (CoRESTs). The SANT (SWI/SNF, ADA, NCoR, and TFIIIB) domain of CoRESTs provides a platform for the assembly of specific transcription inhibitors, and CoRESTs also act as molecular beacons to further attract HDAC1, HDAC2, methyl-CpG-binding protein-2 (MeCP2), and histone H3 and K4 lysine demethylase to promote and maintain methylated CPG-dependent gene silencing (Lakowski et al., 2006; Ooi and Wood, 2007). Under certain conditions, the transcriptional inhibition by NRSF also depends on the synergic repression by other epigenetic regulators, such as C-terminal binding protein, DNA methyltransferase, chromatin remodeling enzyme, Sp3 (one member of the Sp factor family), CDYL (chromodomain on Y-like), MED19, and MED26 (Shi et al., 2003; Mulligan et al., 2008; Ding et al., 2009; Formisano et al., 2015). For example, synergic repression by Sp3 is required for NRSF to suppress ncx1 gene transcription in brain ischemia (Formisano et al., 2015).

Noncoding RNAs, including microRNAs and lncRNAs, as well as ubiquitin and other factors, may be involved in NRSF-based transcription regulation. NRSF represses the expression of many microRNAs, while microRNAs can in turn regulate the expression of NRSF through the regulatory feedback mechanism and participate in the pathophysiological process of the nervous system (Kuwabara et al., 2004; Conaco et al., 2006; Laneve et al., 2010; Hwang et al., 2014; Rockowitz et al., 2014; Brennan et al., 2016). For instance, Laneve et al. (2010) and Conaco et al. (2006) reported that the interaction between miRNA-9 or miRNA-124a and NRSF may be related to the maintenance of neuronal-differentiation programs. Brennan et al. (2016) found that miR-124 is involved in epileptogenesis by effectively blocking NRSF upregulation and enhancing microglia activation and inflammatory cytokines. Ubiquitin is a NRSF regulator that modulates NRSF degradation through ubiquitin-mediated proteolysis via a Skp1-Cul1-F-box protein complex containing an E3 ubiquitin ligase (β-TRCP). Ubiquitin-mediated NRSF degradation is required for proper neuronal differentiation, which promotes the expression of neuron-specific genes in neuronal cells (Westbrook et al., 2008). In addition, other factors, such as nSR100, can also negatively regulate NRSF by inducing alternative slicing of NRSF. The transcripts of NRSF antagonize transcriptional inhibition by NRSF, and thereby activate the expression of NRSF-targeted genes (Raj et al., 2011).

NRSF transport disorder may also be involved in NRSF-mediated transcriptional inhibition. Normally, NRSF is synthesized in the cytoplasm. Most NRSFs need to be transported into the nucleus to bind with NRSE and play a transcriptional-inhibitory role (Zhao et al., 2017). Meanwhile, NRSF retained in the cytoplasm can be drawn to the ribosome to activate translation-initiation factors and upregulate neuron-specific gene expression. However, some special protein molecules, such as the Huntingtin protein, can reduce NRSF in the nucleus by “arresting” part of NRSF in the cytoplasm, thereby upregulating the expression of NRSF target genes in the nucleus (Bessis et al., 1997; Zuccato et al., 2003).

## NRSF and Neurogenesis

At the cellular level, nervous system development is the differentiation of embryonic stem (ES) cell-derived neuronal stem cells into neuronal progenitor cells with a limited self-renewal capacity and then into neuroblasts and glioblasts, which in turn give rise to neurons and glial cells. These changes are macroscopically expressed as the generation of the nervous system (D’Aiuto et al., 2014). Traditionally, the main mechanism of the regulation of nervous system development is believed to be that transcription activators that enhance gene expression are induced by the body step by step to activate neuron-specific gene expression, thus promoting the specialization of the nervous system. However, the advent of NRSF suggested that the “unlocking” of some key genes is also an important regulatory mechanism (Schoenherr and Anderson, 1995a). The release of NRSF from NRSE sequences of these genes results in the release of gene transcriptional inhibition and the expression of characteristic products of the nervous system. To date, these neuron-specific genes containing NRSE are known to include 1) ion channels, such as the NaCh II gene; 2) neurotransmitters and their synthases, such as the neurotransmitter gene *TACl*; 3) synaptic vesicle proteins; 4) cell-adhesion molecules, such as the protocadherin gene; 5) hormones, such as neurokinin B01 and human tyrosine hydroxylase; and 6) neurotrophic factors, cytoskeletal proteins, and extracellular matrix genes (Kraner et al., 1992; Sun et al., 2005; Kim M. Y. et al., 2006; Greco et al., 2007; D’Alessandro et al., 2009; Gillies et al., 2009; Abrajano et al., 2010; Tan et al., 2010). These NRSF-target genes are commonly involved in neuronal differentiation and synaptic plasticity, including ion conductance, axonal growth, and vesicle transport and release (Sun et al., 2005). Under the transcriptional regulation of NRSF, these neuron-specific genes are differentially expressed in various cells at different stages of nervous system development. However, the regulatory mechanism of NRSF synthesis remains unclear (Zhao et al., 2017). Several cytokines and signaling pathways have been shown to regulate NRSF expression, including IκB kinase α, bone morphogenetic protein, Oct4, β-TrCP, canonical Wnt pathways, and RA pathways (Nishihara et al., 2003; Kohyama et al., 2010; Ohnuki et al., 2010; Khoshnan and Patterson, 2012). Intriguingly, the NRSE sequence was also found to be contained in the gene promoter of NRSF, indicating that NRSF expression is also regulated by self-feedback (Sun et al., 2005; Abrajano et al., 2010).

NRSF is involved in the regulation of ES cell totipotency maintenance and self-renewal ability. *In vitro*, the silencing of NRSF target genes in ES cells of mice resulted in the loss of the self-renewal ability of these ES cells, and the expression levels of important genes maintaining cell totipotency, such as Oct4, Nanog, Sox2, Tbx3, and c-myc, were significantly downregulated. Exogenous addition of NRSF protein could restore the self-renewal ability of these cells (Kim S. M. et al., 2006; Singh et al., 2008). *In vivo*, knockout of the NRSF gene in mice resulted in early embryonic lethality with deficient neurogenesis due to the decreased self-renewal ability of ES cells (Zhao et al., 2017). In humans, Bahn et al. (2002) reported that neurological deficits in patients with Down syndrome are due to reduced NRSF expression in ES cells and the premature onset of neuronal differentiation, apoptosis, or neuronal loss. Decreased NRSF levels in ES cells and a mouse model of Down syndrome reduced the expression of totipotency maintenance–related transcription factors such as Oct4, Nanog, and Sox2, while the expression of specific differentiation-related transcription factors (e.g., GATA4, GATA6, FOXA2, PITX2, and SNAI1) were upregulated (Canzonetta et al., 2008). All this evidence suggests that NRSF plays an important role in the self-renewal ability and maintenance of totipotency of the inner cell mass during blastocyst formation. However, several contrary studies have shown that the perturbation of NRSF in embryonic stem cells does not alter their differentiation status (Buckley et al., 2009; Jørgensen et al., 2009; Yamada et al., 2010; Soldati et al., 2012). For example, Jørgensen et al. (2009) reported that NRSF-deficient embryonic stem cells remain pluripotent, capable of differentiating into cells of the three germ layers, i.e., mesoderm, endoderm, and ectoderm. They argued that ES cell pluripotency needs to be evaluated in a complex context, but not only under culture conditions. Moreover, extracellular matrix components, such as feeding cells and laminin, may salvage the role of NRSF in ESC pluripotency (Singh et al., 2012). Taken together, these findings suggest that NRSF is an important but not indispensable element in maintaining ES cell totipotency and self-renewal ability, though NRSF depresses the neuron-specific gene expression program.

NRSF also plays an important part in regulating neuronal differentiation and neurogenesis. *In vitro*, several studies have demonstrated that downregulation of NRSF is required to induce the differentiation of ES cells toward the neuronal lineage (Ekici et al., 2008; Gao et al., 2011). High levels of NRSF in the nucleus of ES cells and neuronal stem cells maintain high transcriptional inhibition of neuron-specific genes, while the level of NRSF in neuronal progenitor cells, neuronal precursor cells, and neurons is gradually decreased, and the expression level of NRSF-target genes such as Drd2, Syt2, and Kirrel3, is gradually increased on the whole, thereby ensuring the normal process of neuronal differentiation and endowing neuronal specificity (Sun et al., 2005). These findings are similar to those of studies by Gupta et al. (2009) and Yang et al. (2008), which found that NRSF regulates the differentiation of ES cells or bone marrow-derived mesenchymal stem cells into neurons, accompanied by the increased expression of various characteristic proteins of neurons. Additionally, several *in vivo* studies also confirmed the roles of NRSF in neuronal differentiation and neurogenesis (Chen et al., 1998; Olguín et al., 2006; Gao et al., 2011). In Xenopus embryos, NRSF inhibition resulted in abnormal neurogenesis, including perturbations of the cranial ganglia, neural tube, and visual development; reduced expression of neural crest markers; and expression loss of pro-neural, neuronal, and neurogenic genes (Olguín et al., 2006). In chicken embryos, NRSF inactivation caused repression of neuronal tubulin and several other neuronal genes, while overexpression of NRSF inhibited endogenous target genes and increased the frequency of axon guidance errors (Chen et al., 1998). Similar findings have been reported in zebrafish (Wang et al., 2012). Knockdown of NRSF resulted in gastrulation delay or blockage and subsequent embryo lethality with deficient neurogenesis (Wang et al., 2012). In summary, this evidence both *in vivo* and vitro demonstrates that NRSF-mediated neuron-specific gene repression is an important regulatory mechanism in neuronal differentiation and neurogenesis.

The development of the nervous system is a complex, continuous, and gradual process. Although NRSF-mediated gene repression is an important regulatory mechanism in the establishment and maintenance of neuronal identity, it also requires post-transcriptional downregulation of non-neuronal transcripts, which is modulated by the interaction between NRSF and microRNAs (Yu et al., 2011; Conti et al., 2012). For example, NRSF regulates the expression of miR-124, and miR-124 in turn targets the messenger RNA (mRNA) of small C-terminal domain phosphatase l, leading to reduced expression of small C-terminal domain phosphatase l in differentiated neurons and the downregulation of NRSF transcriptional inhibition, thus participating in cell differentiation and neurogenesis (Conti et al., 2012). It should be emphasized that NRSF is not the sole master regulator responsible for neuronal fate acquisition; instead, NRSF acts as a regulatory hub that mediates multiple levels of neuronal development (Zhao et al., 2017).

## NRSF and Brain Disorders

The above evidence has shown that NRSF is involved in multiple physiological processes of normal brain function. Therefore, overexpression, underexpression, mutation, or abnormal distribution of NRSF may lead to brain dysfunction. The following neurological diseases have been reported to closely correlate with aberrant expression of NRSF: neurodegenerative diseases (e.g., HD, PD, dementia, Down syndrome, and Niemann–Pick type C disease [NPC]), brain injury (e.g., ischemia injury, global ischemia, and stroke-related brain injury), seizures, mental diseases, and other disorders, like alcoholism (Lepagnol-Bestel et al., 2009; Cai et al., 2011; Yu et al., 2013; Henriksson et al., 2014; Hwang and Zukin, 2018; Kawamura et al., 2019). Comprehensive understanding the underlying mechanisms of NRSF and its copartners in these diseases contributes to identifying potential therapeutic targets.

HD is a neurodegenerative disease directly caused by mutations in the Huntington protein (Htt) (Hwang and Zukin, 2018). Under normal physiological conditions, dynactin p150Glued, huntingtin-associated protein 1 (HAP1), REST-interacting LIM domain protein (RILP), and huntingtin form a complex that can interact with NRSF and be involved in the translocation of NRSF into the nucleus, of which HAP1 is responsible for the cellular localization of NRSF in neurons. The wild-type Htt sequesters NRSF in the cytoplasm of mouse striatum neurons, thereby inhibiting its function (Shimojo and Hersh, 2006; Shimojo, 2008). However, the mutant Huntington protein reduces the binding ability of the complex to NRSF, and NRSF is massively transferred to the nucleus (Hwang and Zukin, 2018). The high concentration of NRSF in the nucleus highly inhibits the transcription of protein-coding genes and non–protein-coding genes containing NRSE sequences, thereby resulting in neuronal death (Zuccato et al., 2007; Johnson et al., 2010). For example, brain-derived neurotrophic factor (BDNF), an NRSF-target gene that is essential for neuronal survival, plasticity, and dendritic growth, is repressed in HD due to the high level of NRSF in the nucleus and the formation of the repressor complex on the promoter of BDNF, ultimately resulting in neurodegeneration (Zuccato et al., 2003). NRSF represses the transcription process of genes other than BDNF, which may also involve in the pathophysiological process of HD, such as synaptophysin, synaptosomal nerve-associated protein 25, fibroblast growth factor 1, and mitochondrial ornithine aminotransferase, which are responsible for synaptic activity, immunomodulation, neurotransmitter release, the secretion of neurotransmitters, striatal neuronal survival, and glutamate synthesis (Wong et al., 1982; Zuccato et al., 2007; Soldati et al., 2011). The underlying mechanisms of NRSF in HD are complicated and remain nebulous. On one hand, NRSF may be involved in HD by blocking the expression of these neuronal genes through an epigenetic mechanism. Deacetylation on the promoters of several neuronal genes that encode neuronal proteins responsible for morphogenesis and neurogenesis was increased, such as polo-like kinase and Ras and Rab interactor 1 (Guiretti et al., 2016). Furthermore, the use of HDAC inhibitors improved motor dysfunction and survival due to less neuronal loss in a mouse model of HD (Ferrante et al., 2004; Gardian et al., 2005). Complicatedly, the epigenetic pathway in HD is not only regulated by NRSF but also by other factors, and it may also occur in the absence of NRSF (Pogoda et al., 2021). On the other hand, NRSF may be involved in HD by interacting with microRNAs. For example, miR-9, a microRNA that regulates NRSF expression levels, upregulated NRSF in HD through a negative feedback mechanism (Packer et al., 2008).

PD is another neurodegenerative disease associated with abnormalities in NRSF. In human dopaminergic SH-SY5Y cells treated with neurotoxin 1 (MPP+), the NRSF expression level and nucleo-plasma distribution ratio in cells were changed, resulting in repression of NRSF-target genes and the death of dopaminergic neurons. Meanwhile, alternation of NRSF expression by RNAi techniques reversed cell viability (Yu et al., 2009). The synchronous occurrence of NRSF abnormality and dopaminergic neuron death suggests that NRSF is related to HD. Furthermore, the tyrosine hydroxylase gene, a rate-limiting enzyme of dopamine synthesis, may be an NRSF-target gene and is repressed by NRSF because suppression of NRSF with the HDAC inhibitor promotes tyrosine hydroxylase promoter activity (Kim M. Y. et al., 2006). Meanwhile, Ohnuki et al. (2010) observed that the expression of NRSF correlates with the dysregulation of striatal genes in an MPTP-lesioned monkey model. Taken together, this evidence implies that NRSF-mediated target gene repression is an important mechanism in the development of PD.

During normal aging, NRSF is induced in part by cell non-autonomous Wnt signaling, and targets and suppresses several genes that promote cell death and Alzheimer’s disease (AD) pathology. However, in several dementias, such as AD, frontotemporal dementia, and dementia with Lewy bodies, NRSF is almost absent from the nucleus of cortical and hippocampal neurons, while it is found in autophagosomes together with misfolded proteins, thus leading to the upregulation of NRSF-target genes and causing neurodegeneration, which is consistent with the finding that conditional deletion of NRSF from the mouse brain leads to age-related neurodegeneration (Lu et al., 2014). NRSF is also involved in NPC, a lysosomal storage disorder-related neurodegenerative disease characterized by cholesterol accumulation in late endosomes and lysosomes, which is caused by a null mutation in the NPC1 gene (Du et al., 2015). In NSCs derived from NPC1-deficient mice, valproic acid (VPA) increases neuronal differentiation and restores impaired astrocytes. Moreover, several neurotrophic genes, such as TrkB, BDNF, and NeuroD, are upregulated by blocking the function of NRSF with VPA treatment (Kim et al., 2007).

The above evidence confirms the involvement of NRSF in the development of neurodegenerative diseases. NRSF is involved in seizures and other epilepsy-promoting insults as well. Increased expression of NRSF in hippocampal neurons has been observed, and the epileptic phenotype can be attenuated by suppressing NRSF function (McClelland et al., 2011). Many NRSE-containing genes in the hippocampus that code for receptors, ion channels, and other important proteins can be restored by blocking the binding site of NRSF to chromatin, which further demonstrates the involvement of NRSF in the development of seizures (McClelland et al., 2014). Furthermore, brain ischemia, global ischemia injury, ischemia–reperfusion injury, or stroke-related injury that causes neuronal damage or delayed death of hippocampal CA1 pyramidal neurons may also be associated with aberrant expression of NRSF. On the one hand, increased NRSF expression in ischemia injury may suppress GluR2 promoter activity and gene expression, which is essential for synaptic plasticity and synaptic remodeling. In an *in vitro* model, Calderone et al. (2003) demonstrated that acute knockdown of the NRSF gene reverses GluR2 suppression and rescues post-ischemic neurons from ischemia-induced cell death. On the other hand, increased NRSF may downregulate mu opioid receptor 1 (MOR-1) mRNA and protein expression which is abundantly expressed in basket cells and inhibitory interneurons of CA1 *via* epigenetic modifications. Ischemia promotes the deacetylation of core histone proteins H3 and H4 and the dimethylation of histone H3 at lysine-9 over the MOR-1 promoter. Acute knockdown of MOR-1 gene expression protects against ischemia-induced death of CA1 pyramidal neurons *in vivo* and *in vitro* (Formisano et al., 2007). In recent research, Luo et al. (2020) reported that cerebral ischemia–reperfusion injury caused a downregulation of HCN1 expression by enhancing the nuclear NRSF–HDAC4 gathering that contributes to neuron damage, which further demonstrates the involvement of NRSF in brain ischemia injury. Additionally, the interaction of NRSF and casein kinase 1 (CK1) or miR-132 (a microRNA that is important for synaptogenesis, synaptic plasticity, and structural remodeling) is also implicated in ischemia-induced hippocampal cell death. Ischemic insults promote NRSF binding and epigenetic remodeling at the miR-132 promoter and silencing of miR-132 expression in selectively vulnerable hippocampal CA1 neurons. Depletion of NRSF by an RNAi technique blocks ischemia-induced loss of miR-132 in insulted hippocampal neurons *in vivo*, consistent with a causal relationship between the activation of NRSF and silencing of miR-132 (Wu and Xie, 2006; Hwang et al., 2014). In addition, overexpression of miR-132 in primary cultures of hippocampal neurons or delivered directly into the CA1 of living rats by means of the lentiviral expression system prior to induction of ischemia protects against ischemia-induced neuronal death (Hwang et al., 2014). Brain ischemia injury also triggered a downregulation of CK1 and an upregulation of NRSF in rat hippocampal CA1 neurons. Administration of the CK1 activator immediately after ischemia could successfully suppress the expression of NRSF and rescue neuronal death (Kaneko et al., 2014). In addition, several studies also revealed the involvement of NRSF in mental diseases. Tateno et al. showed that ethanol-induced neuronal loss is associated with increased NRSF. The abnormal neuronal differentiation of NSCs induced by ethanol can be rescued by lithium and mood-stabilizing drugs by blocking the binding activity of NRS to chromatin (Tateno et al., 2006; Tateno and Saito, 2008).

## NRSF and Tumorigenesis

NRSF plays different roles in different tumor types. Under normal physiological conditions, NRSF is highly expressed in non-neuronal cells, but there is almost no or very low expression of it in mature neuronal cells (Song et al., 2015; Zhao et al., 2017). On the contrary, NRSF expression is decreased in non-neural tumors and increased in neural tumors. Thus, NRSF is thought to play a tumor-suppressor role in non–nervous system tumors, while it acts as a proto-oncogene in nervous system tumors (Huang and Bao, 2012). Currently, the neurogenic tumors that have been reported to be related to NRSF mainly include gliomas, medulloblastomas, and pheochromocytomas, among which adverse drug reactions, high recurrence rate, and poor prognosis are still important problems for patients with such tumors. In this section, we focus on the progress made in the regulatory mechanisms of NRSF in neural tumors.

Glioma (neuroglioma) is the most common type of tumor of the central nervous system and has no effective therapies due to its poor differentiation, rapid proliferation, and strong tissue invasion (Reifenberger et al., 2017). NRSF was found to be highly expressed in glioma tumor cells and tissues. Conti et al. (2012) reported that the expression level of NRSF in tumorigenic glioma cell lines was significantly higher than that in brain-derived neural stem cells. Furthermore, the expression level of NRSF mRNA in glioma tumor tissues was two to five times higher than that in normal brain tissues (Conti et al., 2012). *In vitro*, NRSF was found to be involved in glioma cell line formation. Overexpression of NRSF prevents neuronal cells from differentiating into glial cells but induces glioma cell line formation (Bergsland et al., 2014). By altering the expression of telomere-binding protein 2 and ubiquitin ligase E3, the upstream regulators of NRSF, the growth of glioma stem cells was accelerated, and differentiation was reduced after the reduction in NRSF expression (Bai et al., 2014). Moreover, NRSF is a master regulator that maintains glioblastoma cell proliferation and migration. Inhibition of NRSF suppresses proliferation and migration in glioblastoma cells, partly through regulating the cell cycle by repressing downstream genes (Conti et al., 2012). In clinical practice, Wagoner and Roopra (2012) observed that glioblastoma patients with high NRSF expression have greater malignancy, less sensitivity to chemotherapy, and significantly lower overall survival than patients with low NRSF expression. Taken together, this evidence suggests that high expression of NRSF found in both glioma tissues and cell lines may regulate the growth of glioma cells by affecting their proliferative ability and tumorigenicity, and that NRSF may be a prognostic factor of glioma because the degree of deterioration of glioma is positively correlated with the level of NRSF expression. The underlying mechanisms of NRSF gliomas are complicated. On the one hand, NRSF can regulate the tolerance of glioma cells to glutamate by inhibiting GluR2 expression and improve their adaptability to the living environment. Glutamate secreted by glioma cells can produce a certain degree of cytotoxicity to itself through glutamate receptors on the cell surface, and interference with the expression of glutamate receptors can reduce the sensitivity of glioma cells to glutamate (Ekici et al., 2012). Ekici et al. (2012) reported that the overexpression of NRSF reduced the mRNA expression of the GluR2 gene (an important type of glutamate receptor subtype, containing an NRSF sequence), and the expression of GluR2 increases following interference in NRSF expression with shRNA or induction of NRSF mutation. On the other hand, the actions of NRSF in gliomas involve microRNAs. These microRNAs include tumor-promoting miR-21, miR-10b, and miR26a, and tumor-inhibiting miR-326, miR-128, miR-181, miR-7, and miR-124a (Fu et al., 2018; Huang et al., 2019; Bautista-Sánchez et al., 2020; Bhere et al., 2020; Rezaei et al., 2020; Stakaitis et al., 2020; Wang et al., 2020). For example, high NRSF expression in glioblastoma decreases the miR-124a expression, and thereby increasing the expression of NRSF-target genes, such as SNAI-1 (a transcription factor that promotes cell invasion and tumor metastases), Scp1, and PTPN12 (two small phosphatases), and finally stimulating cell proliferation (Tivnan et al., 2014). In addition, several studies have also confirmed the role of the balance between NRSF and REST4 in gliomas (Chen and Miller, 2013; Ren et al., 2015; Li et al., 2017). REST4 is a truncated transcript of NRSF that can affect the expression of NRSF *in vivo* and *in vitro* (Zhao et al., 2017). Chen and Miller (2013) observed selective shearing of NRSF in a variety of tumor tissues and cell lines, and found that pioglitazone, a peroxidase growth factor activator receptor 1 agonist, can induce selective shearing of NRSF to form REST4, resulting in the loss of an important part of nuclear translocation and preventing NRSF from binding to DNA. In addition, pioglitazone can inhibit the proliferation and induce apoptosis of glioma cells and has an anti-glioma effect, which may be related to the reduction of NRSF expression level regulated by REST4 (Ren et al., 2015).

Medulloblastoma (MB) is the most malignant glioma in the brain. In clinical practice, human MB cells showed high expression of NRSF compared to neuronal precursor cells (NTera2) and fully differentiated human neural cells (hNT), and MB patients with high NRSF expression had poorer overall survival and progression-free survival than patients with low NRSF expression (Lawinger et al., 2000; Taylor et al., 2012). Moreover, the proliferation of MB cells was decreased and apoptosis was increased when transfected with REST–VPL6, a recombinant transcription factor that is a competitive inhibitor of NRSF (Lawinger et al., 2000). These findings were consistent with the finding by Fuller et al. that NRSF was highly expressed in 17 of 21 MB tissues, while NRSF was not expressed in the adjacent tissues, and the growth of MB cells and tissue in the brains of mice was stopped by the use of REST–VPL6, competitively antagonizing the action of NRSF (Fuller et al., 2005). Taken together, this evidence suggests the involvement of NRSF in MB. However, the underlying mechanism of NRSF in MB is complicated and remains elusive. *In vitro*, sertraline, chlorprothixene, and chlorpromazine inhibit MB cell growth by acting with the NRSF-binding site of the corepressor mSin3 (Kurita et al., 2018). Furthermore, knockdown of NRSF, which is highly expressed in MB cells, can relieve the inhibitory effect on the gene encoding the de-ubiquitination enzyme USP37, upregulate the expression of USP37 protein and promote the de-ubiquitination of tumor suppressor p27 and stabilize its expression, thus inhibiting the proliferation of tumor cells, which indicates that the classical action of NRSF, namely NRSF-mediated transcription inhibition—especially the NRSF-USP37-P27 pathway—may be a vital regulatory mechanism in the development of MB (Das et al., 2013). Additionally, NRSF may also influence the proliferation and pathogenicity of MB through other mechanisms. For example, increased NRSF promotes the growth of MB, possibly by promoting vascular growth through autocrine and paracrine mechanisms, which further demonstrates the complexity of the NRSF regulatory mechanism in MB (Shaik et al., 2021).

In summary, various studies have implicated the oncogenic role of NRSF in neural tumors, and several NRSF-mediated regulatory mechanisms of tumorigenesis have been identified. However, further studies are warranted to reveal the comprehensive cell biological networks of tumorigenesis by which NRSF governs cell proliferation, cell transformation, and tumor growth.

## NRSF and Neuropathic Pain

NPP is a common chronic pain in clinical practice that is mainly caused by sensitization of the central or peripheral nervous system. It is widely considered one of the most difficult pain syndromes to treat, and current therapeutic strategies are largely ineffective due to a lack of understanding of its causes (Finnerup et al., 2021). In recent years, several studies have shown NRSF-mediated suppression of specific genes is an important component in the development and maintenance of NPP.

The Kcnd3 gene encodes the Kv4.3 channel protein, which is widely involved in the process of sensory signal transduction. In the Kcnd3 genes of mouse, rat, and human, a conserved NRSE sequence was verified. Using a mouse sciatic nerve injury model, (Uchida et al., 2010a), found that NRSF expression was upregulated and Kcnd3 expression was downregulated, with the binding of NRSF to NRSE in the promotor region of Kcnd3 significantly increased and histone H4 acetylation decreased. Anti-sense nucleotide knockdown of NRSF can reverse the downregulation of Kv4.3 channel expression, which further demonstrates that NRSF plays important roles in modulating the expression of Kv4.3 and the development and maintenance of NPP (Uchida et al., 2010b). In the Scn10a gene, similar regulatory mechanisms were observed. The expression of NRSF in the dorsal root ganglion (DRG) is increased after nerve injury, and it specifically binds to NRSE sequence in the promoter region of the Scn10a gene and downregulates the transcription and ion channel protein expression through an epigenetic mechanism, thereby participating in the primary sensory nerve C-fiber inactivation process. Knockdown of NRSF with the HDAC inhibitor trichostatin A, VPA, suberoylanilide hydroxamic acid or anti-sense oligonucleotide can effectively reverse the downregulation of Scn10a transcription and the inhibition of C fiber function (Uchida et al., 2010a). Our previous work in a sarcoma murine model verified that MOR mRNA expression is also regulated by NRSF. In DRG neurons of sarcoma-bearing mice, NRSF expression is upregulated while MOR mRNA expression is downregulated, accompanied by promoted binding of NRSF to NRSE within the promoter area of the MOR gene and a hypoacetylation state of histone H3 and H4. Genetically knocking down NRSF with anti-sense oligodeoxynucleotide rescued the expression of MOR and potentiated the analgesic effect of morphine (Zhu et al., 2017). These findings were also demonstrated in a nerve injury pain model, which further indicates that NRSF-mediated transcription inhibition of the MOR gene is an important regulatory mechanism in pain signal transduction (Kim et al., 2004). Chrm2 is another gene that is selectively repressed by NRSF in NPP. Zhang et al. (2018) reported that nerve injury persistently increased the NRSF expression and reduced Chrm2 expression in DRG neurons, as well as increasing the enrichment of NRSF in the Chrm2 promoter and diminishing the analgesic effect of muscarine. Knockdown or genetic ablation of NRSF in DRG neurons rescued the Chrm2 expression and augmented muscarine’s analgesic effect on NPP (Zhang et al., 2018). Currently, the NRSF-mediated transcriptional inhibition target gene pool has not been well established in NPP. Many genes upregulated in the microarray and RNA sequencing studies of NPP mouse models also have promoter regions that show occupancy by NRSF or its cofactors (such as SIN3A, CoREST, and HDAC1/2) in the ENCODE database. These genes include Cacna2d1 (an L-type voltage-gated calcium channel), GABAA receptor 5 subunit, vasoactive intestinal peptide, growth-associated protein 43, and Gadd45a (DNA demethylase) (Perkins et al., 2014). Further study is warranted to verify whether these genes are involved in NPP in the future. Taken together, these findings indicate that NRSF regulates the gene transcription of various nociceptive transduction molecules through epigenetic mechanisms to affect the development and maintenance of NPP.

The epigenetic mechanisms of NRSF-mediated selective gene repression in NPP remain elusive. On the one hand, in the same way as its classical action, NRSF recruits co-repressors, such as mSin3 and HDACs, and then deacetylates nucleosome histones, thereby inhibiting the transcription of target genes involved in NPP. In a diabetic pain model, Xiao-Die et al. (2020) reported that high glucose and high palmitic acid treatment induced the upregulation of NRSF and its cofactors (mSin3A, CoREST, and HDAC1) but the downregulation of GluR2 and NMDAR2B in the anterior cingulate cortex neurons. Knockdown of NRSF could partially reverse the expression changes of HDAC1 and NMDAR2B. These findings were further confirmed by Ueda et al. (2017), who found that a chemically optimized mimetic mS-11 (a mimetic of the mSin3-binding helix) can inhibit mSin3–NRSF binding and successfully reverse lost peripheral and central morphine analgesia in mouse models of pain. In addition, previous studies also have shown that HDAC inhibitors reduce pain symptoms in a variety of pain models by eliminating NRSF-mediated chromatin remodeling and transcriptional inhibition (Bai et al., 2015). On the other hand, pain-induced phosphorylation of MeCP2 may be another potential epigenetic mechanism for NRSF-mediated gene transcription inhibition in NPP. MeCP2 is a methylated DNA-binding protein that recruits co-inhibitory complexes and promotes NRSF recruitment of CoREST (Chen et al., 2003). In a mouse model of pain, elevated phosphorylation of MeCP2 was observed in DRG neurons. When MeCP2 is phosphorylated, its affinity for methylated DNA is reduced, and the transcription levels of its target genes are thereby promoted (Géranton et al., 2007). Additionally, whether the actions of NRSF in NPP involve microRNAs remains controversial, though several studies have documented decreased microRNA expression in chronic pain models (Descalzi et al., 2015). For example, expression levels of miR-7a, -134, and -96 were downregulated in the DRG neurons in various rodent pain models (Ni et al., 2013; Sakai et al., 2013; Chen et al., 2014). However, the promoter regions of these downregulated microRNAs do not contain NRSE or NRSF-related binding sequences (Willis et al., 2016). There are two possible explanations for the contrary phenomenon (altered expression but without NRSE sequences). First, the downregulation of these microRNAs may not be mediated by NRSF but instead by other inhibitory factors. Second, the decreased microRNAs may be modulated by distant NRSF complexes, because previous studies have demonstrated that several microRNAs are regulated by NRSF in several physiological or pathological processes of the nervous system. In the future, further studies are required to investigate the upregulation mechanism of NRSF expression and the molecular mechanism of NRSF regulation and inhibition of the transcription level and post-transcription level of target genes, as well as the roles of alternative splicing and microRNAs in NPP.

## Clinical Prospects

The abovementioned findings highlight the master role of NRSF not only in modulating neurogenesis by dynamically inhibiting neuron-specific genes expression, but also in certain brain disorders. It hence provides a potential therapeutic target for these disorders, which has attracted significant attention over the past few decades, especially regarding neurodegenerative diseases, epilepsy, and neural tumors. Here, we summarized some potential therapeutic approaches that target NRSF or its co-repressors for such disorders.

Some small molecules, such as 4SC-202 and SP2509, that target the interaction between the C-terminal domain of NRSF and CoREST have been tested (Inui et al., 2017). Such small molecules could inhibit the deacetylase and demethylase activity of the NRSF -CoREST complex in MB cells and negatively affected cell viability (Inui et al., 2017). Many drugs focusing on restoring the homeostasis of NRSF, such as X5050 or REST-VP16, may be more promising candidates for NRSF-related brain diseases (Su et al., 2004; Charbord et al., 2013). X5050, a chemical compound that targets NRSF degradation, can upregulate the expression of certain neuronal genes though promoting NRSF degradation. This was demonstrated in an HD model in which treatment with X5050 increased the expressions of BDNF and several other NRSF-regulated genes by degrading increased NRSF in the nucleus (Charbord et al., 2013). REST-VP16, a competitive antagonist of NRSF, can bind to the same DNA binding site as NRSF does. However, it functions as an activator instead of a repressor and can directly activate the gene transcription suppressed by NRSF. This mechanism has been verified by both *in vitro* and *vivo* studies (Su et al., 2004). In an HD model, heat shock protein 90 (Hsp90) was reported necessary to maintain the levels of NRSF and huntingtin proteins. Inhibition or knockdown of Hsp90 reduced the levels of NRSF and mutant huntingtin in the nucleus and rescued cells from mHtt-induced cellular cytotoxicity, thus providing neuroprotective activity (Orozco-Díaz et al., 2019). These findings suggest that rescuing the aberrant expression of NRSF is a promising approach for the related central nervous system disorders. However, much work is still needed to further investigate the underlying mechanisms as well as the accompanying side effects of such drugs. As there are hundreds of targets of NRSF, it may trigger distinct cellar pathways in different neurological disorders to exert its multiple roles.

## Conclusion

NRSF acts as a master regulator in the pathophysiological processes of the nervous system. A dynamic level of NRSF under physiological conditions is required for proper neurogenesis, while aberrant expression is associated with many brain disorders, such as neurodegenerative diseases and neural tumors. At present, the comprehensive pathogenic mechanisms and promising therapeutic targets based on NRSF remain elusive, though several NRSF-targeting compounds or mimetics are being constructed and tested in various models.

Even though NRSF may be a promising clinical biomarker and treatment target of brain disorders in the future, some major problems remain to be solved. First, though it is one of the most important transcriptional regulators in nervous system development and mediates common signaling pathways in many brain diseases, the mechanism by which NRSF itself is regulated remains unknown. Second, NRSF acts not only as an inhibitor but also an activator, which means that it may trigger distinct cellar pathways in different neurological disorders. Hence, more evidence is required to confirm the comprehensive regulatory mechanism of NRSF. In particular, even though the interaction between NRSF and microRNAs in the nervous system has been reported, the combination and molecular mechanism remain unclear, which may be a novel therapeutic target in the future.
